# Risk factors for shoulder disorders among French workers: prospective cohort study

**DOI:** 10.1007/s00420-022-01853-9

**Published:** 2022-03-16

**Authors:** Julie Bodin, Ronan Garlantézec, Alexis Descatha, Bradley Evanoff, Thierry Thomas, Yves Roquelaure

**Affiliations:** 1 Univ Angers, Univ Rennes, Inserm, EHESP, Irset (Institut de recherche en santé, environnement et travail) - UMR_S 1085, SFR ICAT, F-49000 Angers, France; 2 Univ Rennes, CHU Rennes, Inserm, EHESP, Irset (Institut de recherche en santé, environnement et travail) - UMR_S 1085, F-35000 Rennes, France; 3 Univ Angers, CHU Angers, Univ Rennes, Inserm, EHESP, Irset (Institut de recherche en santé, environnement et travail) - UMR_S 1085, SFR ICAT, F-49000 Angers, France; 4 Inserm, UMS 011, Unité Cohortes Epidémiologiques en Population, Villejuif, France; 5 Division of General Medical Sciences, Washington University School of Medicine, St. Louis, St. Louis, MO 13 63310 USA; 6 Department of Rheumatology, CHU Saint-Etienne, Saint-Etienne, France; 7 INSERM U1059, Université de Lyon, Saint-Etienne, France

**Keywords:** Shoulder disorder, Musculoskeletal, Work, Occupational exposure, Structural equation modelling

## Abstract

**Objectives:**

Shoulder disorders are common in the working population. This longitudinal study aimed to explore the relationships between personal factors and occupational organisational, psychosocial, and biomechanical factors and the incidence of shoulder disorders.

**Methods:**

A total of 3710 workers in the Pays de la Loire region (Loire Valley area, France) were randomly included by their occupational physician in the Cosali cohort between 2002 and 2005. All workers completed a self-administered questionnaire about personal factors and work exposure, and using a standardised physical examination, occupational physicians diagnosed shoulder disorders. Between 2007 and 2010, 1611 workers were re-examined by their occupational physician. The 1,320 workers free of shoulder disorders at baseline were studied. A conceptual model was developed in which relationships between organisational, psychosocial, biomechanical, and personal factors at baseline and the incidence of shoulder disorders were assumed. Structural equation modelling was used to test the model.

**Results:**

Shoulder disorders were directly associated with biomechanical factors and age but not with psychosocial factors. However, skill discretion and psychological demand influenced shoulder disorders indirectly through biomechanical factors. Exposure to a work pace dependent on an automatic rate and to a work pace dependent on customers’ demands were associated with biomechanical and psychosocial factors, but not directly to shoulder disorders.

**Conclusions:**

This study identified the complex direct and indirect relationships between occupational factors and shoulder disorders. Our data confirmed our conceptual causation model: organisational and psychosocial factors were associated with biomechanical factors, while biomechanical factors were associated with the incidence of shoulder disorders.

**Supplementary Information:**

The online version contains supplementary material available at 10.1007/s00420-022-01853-9.

## Introduction

The rotator cuff is made up of the muscle belly and tendons of the infraspinatus, supraspinatus, teres minor, and subscapularis muscles. Shoulder disorders describe any injury or degenerative condition affecting the anatomical structures of the shoulder including the rotator cuff (subacromial impingement syndrome, bursitis, rotator cuff tendonitis, partial or full-thickness rotator cuff tears). Shoulder disorders are common in the general and working population, with prevalence ranging from 2 to 8% (Dalbøge et al. 2019). In France, shoulder disorders accounted for 30% (*n* = 15,241) of occupational diseases in 2019 (Caisse Nationale de l’Assurance Maladie 2020). Shoulder disorders cause long absences from work (Clausen et al. 2021).

Literature has shown the multifactorial origin of shoulder disorders. Some personal factors increase the risk of shoulder disorders (e.g., age, obesity) (Wærsted et al. 2020). In addition, literature reviews have shown low to moderate evidence of an association between shoulder disorders and work-related arm posture and forceful shoulder exertion, and a low level of evidence for psychosocial exposure at work (e.g., high job demands, low job control and low social support) (van der Molen et al. 2017; Dalbøge et al. 2019; Wærsted et al. 2020). Some epidemiological studies have investigated the organisational risk factors for upper-extremity musculoskeletal disorders (UEMSDs), e.g., (Lamy et al. 2014; Widanarko et al. 2014; Liu et al. 2015; Bao et al. 2016; de Kok et al. 2019), but few studies have disentangled the relationships between exposure to work-related biomechanical, psychosocial, and organisational factors and shoulder disorders.

Several conceptual models (Hagberg et al. 1995; Sauter and Swanson 1996; Karsh 2006; MacDonald et al. 2008; Punnett et al. 2009; Stock et al. 2013; Roquelaure 2016) underline the role of factors related to work organisation at the company level (meso level, e.g., production on assembly lines with automatic machine and management practices) in the occurrence of UEMSDs, since these factors influence exposure to psychosocial and biomechanical risk factors at the individual level. Using logistic regression models with workers enrolled in the Cosali cohort, we showed that only one factor related to work organisation was associated with the incidence of shoulder disorders in women, namely, “work with temporary workers” (Bodin et al. 2012). In the case of UEMSDs, conceptual models assume that biomechanical factors are a predictor of UEMSDs and an outcome of organisational factors. The methodological issue with logistic regression is that it does not enable a variable to be studied as a predictor and an outcome. More appropriate statistical models, such as structural equation modelling (SEM), may provide a better understanding of the complex relationships between the factors influencing the risk of shoulder disorders. Some studies have used SEM to investigate the relationships between workplace risk factors and UEMSDs (e.g., Sprigg et al. 2007; Park et al. 2010; Larsman et al. 2013, p. 201; Abdul Rahman et al. 2017; Mehralizadeh et al. 2017), but few have studied the role of organisational factors (Bodin et al. 2018, 2020; Roquelaure et al. 2020).

To improve the prevention of shoulder disorders in the working population, it is important to understand how work organisation influences biomechanical and psychosocial factors, and finally shoulder disorders. The objective of this study was to explore the relationships between occupational—organisational, psychosocial, and biomechanical factors—and personal factors at baseline, and shoulder disorders at follow-up in French workers using SEM.

## Methods

### Conceptual model

**Fig. 1:**
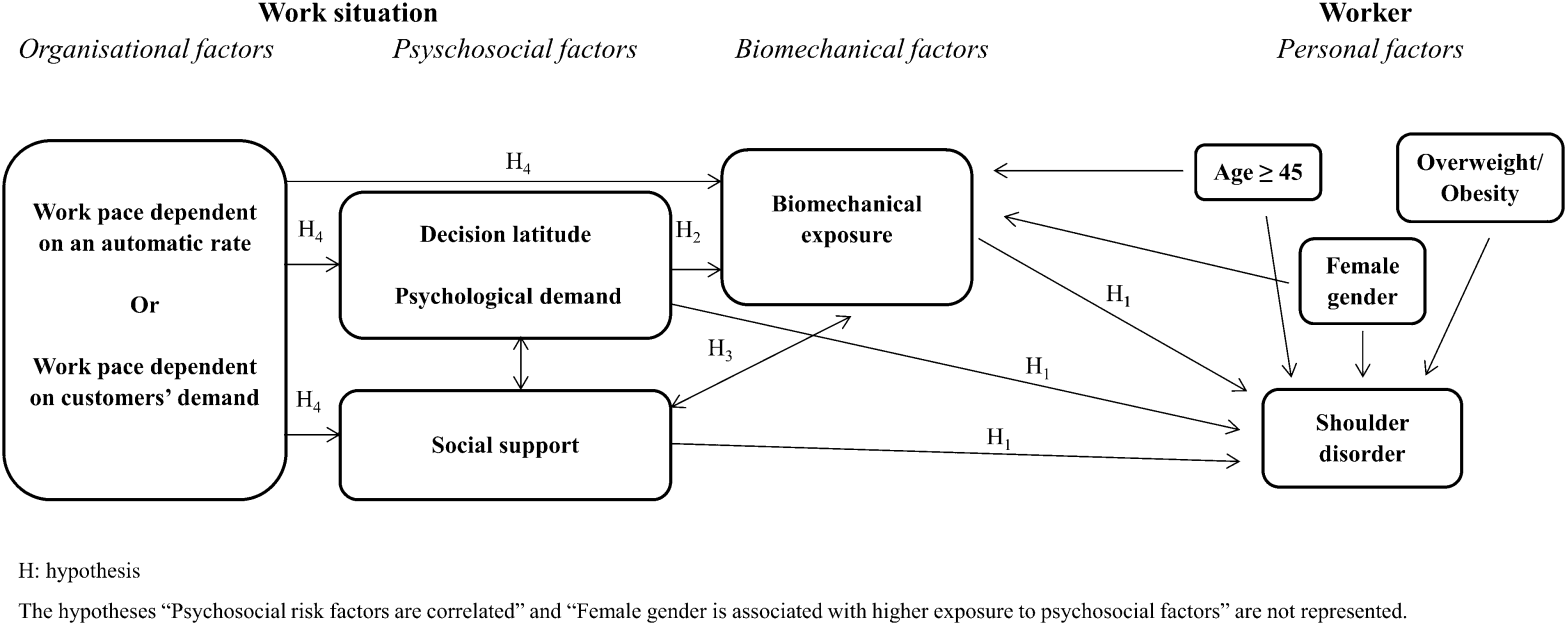
Conceptual model of the relationships between organisational, psychosocial, biomechanical, and personal factors and shoulder disorder

A conceptual model was defined based on the literature (Hagberg et al. 1995; Sauter and Swanson 1996; Karsh 2006; MacDonald et al. 2008; Punnett et al. 2009; Stock et al. 2013; Roquelaure 2016), the authors’ expertise and data available in the sample studied (Fig. 1). This model had already been tested for shoulder pain (self-reported shoulder pain without diagnosis of shoulder disorder confirmed by an occupational physician) (Bodin et al. 2018, 2020) and carpal tunnel syndrome (CTS confirmed by an occupational physician) (Roquelaure et al. 2020) in the same database. The following hypotheses were tested:

1. According to hypothesis 1, the risk of shoulder disorders is directly increased by biomechanical (Hagberg et al. 1995; van Rijn et al. 2010; Mayer et al. 2012; van der Molen et al. 2017; Dalbøge et al. 2019) and psychosocial (van Rijn et al. 2010; van der Molen et al. 2017; Dalbøge et al. 2019) factors.
2. According to hypothesis 2, exposure to psychosocial factors, namely, low decision latitude and high psychological demand, can indirectly influence shoulder disorders by increasing exposure to biomechanical factors (Park et al. 2010; Thiese et al. 2015).
3. According to hypothesis 3, the relationships between social support and biomechanical factors may be twofold (Thiese et al. 2015): (i) exposure to high biomechanical loads may require more cooperation and social support between coworkers to handle the task and reduce biomechanical exposure; (ii) conversely, lack of social support may give workers less opportunity to diminish biomechanical exposure.
4. According to hypothesis 4, exposure to factors related to work organisation, namely, a work pace dependent on an automatic rate, and a work pace dependent on customers’ demands, can influence biomechanical and psychosocial factors (Hagberg et al. 1995; Sauter and Swanson 1996; Karsh 2006; MacDonald et al. 2008; Punnett et al. 2009; Stock et al. 2013; Roquelaure 2016).

Additional hypotheses were tested: (i) psychosocial risk factors are correlated, (ii) age increases the risk of shoulder disorders and reduces exposure to biomechanical risk factors, (iii) overweight/obesity increases the risk of shoulder disorders, (iv) female gender is associated with shoulder disorders, higher exposure to psychosocial factors and lower exposure to biomechanical factors as compared to men.

### Study population

The Cosali cohort study has previously been described in detail (Roquelaure et al. 2006; Bodin et al. 2012, 2018). In short, this prospective study was based on two successive surveys of a large sample of workers in the French Pays de la Loire region. At the time of the first survey, all French salaried workers underwent a mandatory annual health examination by an occupational physician (OP) in charge of the medical surveillance of a group of companies. Between 2002 and 2005, 83 OPs (18% of OPs in the region) from the Pays de la Loire region volunteered to take part in the study. A total of 3,710 workers were selected at random, following a two-stage sampling procedure: first, 15–30 half days of scheduled examinations for each OP were chosen for sampling by the investigators. Next, each OP was asked to randomly select from the schedule 1 of 10 workers on the selected half days of worker examinations. Workers aged between 20 and 59 years of age, working in the Pays de la Loire region, regardless of their type of employment contract, and under surveillance by the 83 OPs were eligible for inclusion. Medical follow-up of 1,611 initially included workers was undertaken between 2007 and 2010 (Bodin et al. 2012). Supplementary Appendix 1 presented a comparison between the 1611 workers with a follow-up and the 2099 workers without follow-up.

Workers were excluded from analyses if they: (i) were craftsmen, salesmen, and managers and workers in the agriculture sector at baseline because of the low number of subjects in these occupations and economic sectors (*n* = 22), (ii) had shoulder disorders at baseline (*n* = 148), and (iii) had missing data for at least one of the variables studied (*n* = 121). A total of 1320 workers (775 men and 545 women) were included in the study (Supplementary Appendix 2).

### Measurements at baseline

At baseline, workers completed a self-administered questionnaire about sociodemographic factors, musculoskeletal pain, and their working conditions during a typical working day over the preceding 12 months.

Two factors related to work organisation were considered (yes/no): a work pace dependent on customers’ demands and a work pace dependent on an automatic rate. The latter was established by two questions: “During a typical day, is your work pace imposed by the automatic rate of a machine?” and “During a typical day, is your work pace imposed by the automatic movement of a product or item?”, if the worker responded “yes” to at least one of the questions, the worker was considered to be exposed. Psychosocial work factors were assessed using the French version of Karasek’s Job Content Questionnaire (JCQ) (Niedhammer et al. 2006). The dimensions were studied as continuous: decision latitude (i.e., decision authority and skill discretion), psychological demand, and social support (i.e., supervisor social support and co-worker social support). Biomechanical factors were selected according to the European consensus criteria to access the work-relatedness of UEMSDs (Sluiter et al. 2001) and were also based on previous results using the same study data (Roquelaure et al. 2011; Bodin et al. 2012). Factors included working with arms abducted at ≥ 60° (never or practically never or rarely (< 2 h/day) vs. often (2–4 h/day) or always (> 4 h/day)), working with arms at or above shoulder level (never or practically never or rarely (< 2 h/day) vs. often (2–4 h/day) or always (> 4 h/day)), and perceived physical exertion (Borg Rating Perceived Exertion (RPE) scale graded from 6 (“no exertion at all”) to 20 (“maximum exertion”) and studied as continuous). Age was dichotomised at 45 years (Djade et al. 2019). BMI was divided into two categories, using the World Health Organization criteria: underweight/normal weight (< 25 kg/m^2^) and overweight/obese (≥ 25 kg/m^2^).

### Shoulder disorder at follow-up

Shoulder disorders were assessed in the same way at baseline and at follow-up. In cases of shoulder pain occurring during the preceding 12 months, a physical examination was performed by the OP using a standardised clinical procedure (Sluiter et al. 2001). Shoulder disorder was diagnosed if (i) there was at least intermittent pain in the shoulder region (without paraesthesia), worsened by active elevation movements of the upper arm, as in scratching the upper back, currently or for at least 4 days during the preceding 7 days; and (ii) if at least one of the following shoulder tests was positive: resisted shoulder abduction, external or internal rotation; resisted elbow flexion (palm-up test); painful arc on active upper arm test (abduction-elevation).

### Statistical analyses

Pearson’s chi-squared test for nominal variables and Student’s *t* test for continuous variables were used to compare the workers’ characteristics according to shoulder disorder.

The relationships between exposure variables and incident shoulder disorders established in the conceptual model (Fig. 1) were assessed using SEM in the whole sample of workers (Beran and Violato 2010). In SEM, two types of variables are defined: manifest and latent variables. Manifest variables (represented by rectangles in the path diagram) are observed variables, whereas latent variables (represented by circles) are variables that cannot be measured directly (biomechanical here) and are estimated through the observed variables (arms abducted at ≥ 60°, working with arms at or above shoulder level and perceived physical exertion). SEM was performed with the lavaan package of R software v3.6.1 using the WLSMV estimator (weighted least squares estimation with robust standard errors and a mean- and variance-adjusted test statistic) (Finney and DiStefano 2013). Standardised beta parameters (interpretable in terms of correlation and ranging from − 1 for a perfect negative association to 1 for a perfect positive association) were presented and statistical significance was defined as a p-value lower than 0.05. A Tucker-Lewis index (TLI) and a comparative fit index (CFI) greater than 0.90, a root mean square error of approximation (RMSEA) of 0.08 or below, and a ratio of χ^2^ to the degree of freedom of 3 or less indicated good fit for the model (Hoe 2008).

## Results

A total of 86 cases of shoulder disorder (6.5%) were diagnosed at follow-up (46 in men (5.9%) and 40 in women (7.3%), *P* = 0.309). Mean follow-up after the first examination was 5.4 (standard deviation = 1.2, range: 2.9–9.0 years). Workers with shoulder disorder were older, overweight, or obese and worked more often with arms above shoulder level than workers without shoulder disorder (Table 1).

**Table 1:** Comparison of worker characteristics according to shoulder disorder (*n* = 1320)

	No shoulder disorder (*n* = 1234)				Shoulder disorder (*n* = 86)				*P* value
	*n*	%	Mean	Standard deviation	*n*	%	Mean	Standard deviation	
Age ≥ 45	331	26.8			45	52.3			** < 0.001**^a^
Overweight/obesity	437	35.4			41	47.7			**0.022**^a^
Work pace dependent on an automatic rate	171	13.9			13	15.1			0.745^a^
Work pace dependent on customers’ demand	573	46.4			42	48.8			0.666^a^
Arms above shoulder level (≥ 2 h/day)	122	9.9			15	17.4			**0.026**^a^
Arms abducted (≥ 2 h/day)	179	14.5			18	20.9			0.106^a^
Perceived physical demand			11.5	3.1			12.2	3.2	0.062^b^
Decision authority			36.3	7.0			35.5	8.1	0.322^b^
Skill discretion			34.4	6.3			34.6	5.9	0.740^b^
Job demand			21.4	3.6			21.9	3.9	0.300^b^
Supervisor social support			11.6	2.2			11.3	2.0	0.215^b^
Coworkers social support			12.6	1.8			12.4	1.8	0.364^b^

^a^Chi^2^ test comparing baseline characteristics according to presence or not of shoulder disorder

^b^Student's *t* test comparing baseline characteristics according to presence or not of shoulder disorder

In bold, *p* value < 0.05

**Fig. 2:**
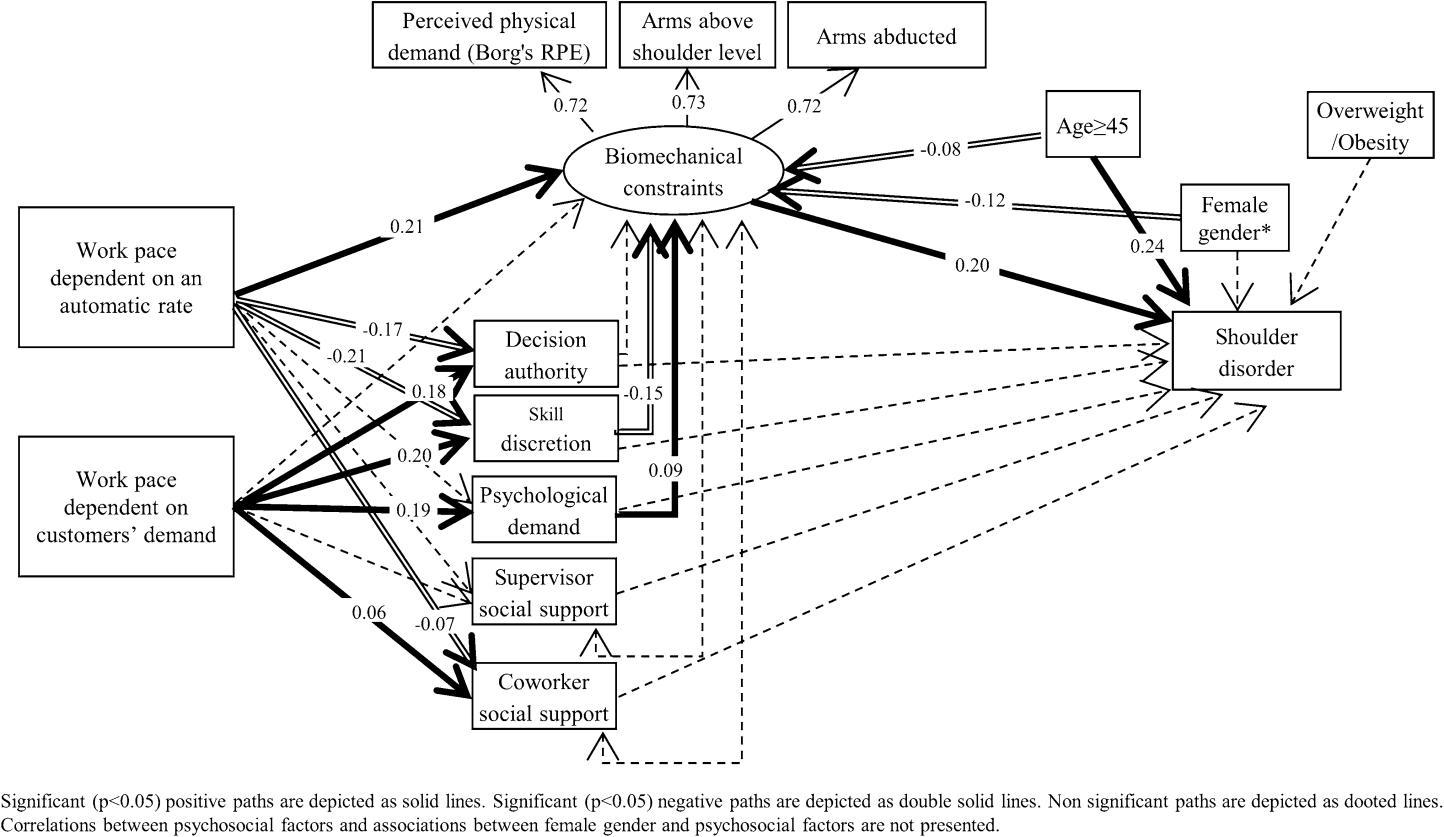
Structural equation model of the relationships between organisational, psychosocial, biomechanical, and personal factors and shoulder disorder in French workers, Cosali (COhorte des SAlariés Ligériens) survey (*n* = 1320)

The model fit was good (*X*^2^/degrees of freedom ratio = 1.3, RMSEA = 0.01 (95% CI 0.00–0.03), CFI = 0.96, TLI = 0.99, SRMR = 0.03). Shoulder disorders were directly associated with biomechanical factors (standardised coefficient sc = 0.20, *p* = 0.004) and none of the psychosocial factors were directly associated with shoulder disorders (Fig. 2 and Supplementary Appendix 3. However, high skill discretion was associated with lower exposure to biomechanical factors (sc = −0.15, *p* < 0.001), whereas high psychological demand was associated with higher exposure to biomechanical factors (sc = 0.09, *p* = 0.005). For organisational factors, exposure to a work pace dependent on an automatic rate was indirectly associated with shoulder disorders, by increasing the exposure to biomechanical factors (sc = 0.21, *p* < 0.001) and decreasing the skill discretion (sc = −0.21, *p* < 0.001), which in turn was associated with biomechanical factors. An automatic rate was also negatively associated with decision authority (sc = −0.17, *p* < 0.001) and coworkers’ social support (sc = −0.07, *p* = 0.007). Exposure to a work pace dependent on customers’ demands had the opposite effect, by increasing decision authority (sc = 0.18, *p* < 0.001), skill discretion (sc = 0.20, *p* < 0.001), psychological demand (sc = 0.19, *p* < 0.001) and coworkers’ social support (sc = 0.06, *p* = 0.027).

Age was directly associated with shoulder disorders (sc = 0.24, *p* < 0.001) and also indirectly associated with shoulder disorders by being associated with lower biomechanical factors (sc = −0.08, *p* = 0.027). Female gender and overweight/obesity were not directly associated with shoulder disorders. However, female gender was associated with lower exposure to biomechanical factors, decision authority and skill discretion.

Regarding the analyses stratified by gender, the results were the same overall (supplementary Appendix 3): (i) a direct effect of biomechanical factors on the risk of shoulder disorders in both genders (sc = 0.17, *p* = 0.051 in men and 0.20, *p* = 0.023 in women), and no direct effect for psychosocial factors, although the association between psychological demand and shoulder disorders was of borderline significance (sc = 0.13, *p* = 0.052). The results also showed a work pace dependent on an automatic rate to have an indirect effect on shoulder disorders by increasing biomechanical factors; ageing was found to have a direct effect. In both genders, an automatic rate was also negatively associated with decision authority and skill discretion, while exposure to a work pace dependent on customers’ demands had the opposite effects, by increasing decision authority, skill discretion and psychological demand. However, the association between skill discretion and biomechanical factors was only observed in women, while the association between psychological demand and biomechanical factors was only observed in men. A negative effect of a work pace dependent on an automatic rate on supervisor and coworkers’ social support was only statistically significant in women, and a positive effect of a work pace dependent on customers’ demands on coworkers’ social support was only statistically significant in men.

## Discussion

Using structural equation modelling, which enabled the complex relationships between variables to be investigated, this study showed that organisational and psychosocial factors were associated with biomechanical factors, while biomechanical factors were linked to the incidence of shoulder disorders.

The study confirmed previous results, using the same database and logistic regression (Bodin et al. 2012), showing that biomechanical factors were a major risk factor for shoulder disorders (hypothesis 1). This is consistent with several literature reviews on shoulder disorders (van Rijn et al. 2010; Mayer et al. 2012; van der Molen et al. 2017; Dalbøge et al. 2019) and with epidemiological studies using SEM (Park et al. 2010; Mehralizadeh et al. 2017). However, Wærsted et al. found limited evidence for an association between shoulder disorders and arm elevation at work and moderate evidence for severe arm elevation with elbows above shoulder level (i.e., > 90°) (Wærsted et al. 2020).

Our results showed an indirect influence of psychosocial factors on shoulder disorders through biomechanical factors which in turn influenced the risk of shoulder disorders. Indeed, skill discretion was negatively associated with biomechanical factors, and psychological demand was positively associated with these factors (hypothesis 2). This is consistent with epidemiological literature (Park et al. 2010; Roquelaure et al. 2020). No direct relationships were observed between psychosocial factors and shoulder disorders, contrary to some epidemiological studies using SEM (Park et al. 2010; Mehralizadeh et al. 2017). In addition, the study did not show any correlations between biomechanical factors and social support (hypothesis 3). Several studies have shown an indirect effect of psychosocial factors on UEMSDs or musculoskeletal pain through anxiety (Sprigg et al. 2007), depression (Sprigg et al. 2007), stress (Larsman et al. 2013) or work-family conflict (Abdul Rahman et al. 2017). In a previous study, we showed that psychological demand was associated with shoulder pain via perceived stress (Bodin et al. 2018). This association cannot be excluded although it could not be tested in this study, because perceived stress was assessed several years before shoulder disorders was diagnosed.

As mentioned in previous studies in the same database (Bodin et al. 2018, 2020; Roquelaure et al. 2020), the study showed that organisational factors influence biomechanical and psychosocial factors. Indeed, a work pace dependent on an automatic rate (e.g., machine-paced jobs) was associated with biomechanical factors. Such organisation is characteristic of blue-collar workers. Previous results on workers from the industrial sector, mainly blue-collar workers, showed the same results (Bodin et al. 2020). Our results are in line with the ergonomics literature showing that automated production lines tends to increase biomechanical exposures (Westgaard and Winkel 2011; St-Vincent et al. 2014; Bao et al. 2016). In addition, in our study, a work pace dependent on an automatic rate was also associated with psychosocial factors, by decreasing decision authority, skill discretion, and coworkers’ social support. This is consistent with Melin et al*.* (Melin et al. 1999) which showed that flexible work organization (small autonomous groups having greater opportunities to influence the pace and content of their work) induces less stress than the assembly line.

Conversely, a work pace dependent on customers’ demands (e.g., cashiers’ work) was associated with higher skill discretion, which in turn decreased the exposure to biomechanical factors and thereby decreased the risk of shoulder disorders. A work pace dependent on customers’ demands was also associated with higher psychological demand, therefore, leading to an increased risk of shoulder disorders via the influence on biomechanical factors. In addition, in our study, a work pace dependent on customers’ demands was also associated with higher decision authority and higher coworkers’ social support. Work pace dependent on customers’ demands is characteristic of professionals, technicians and lower grade white-collar workers and wholesale and retail trade. Few epidemiological studies have investigated associations between a work pace dependent on customers’ demands and psychosocial factors (Melin et al. 1999).

With regard to personal factors, the study showed that age increased the occurrence of shoulder disorders, in agreement with the literature (Djade et al. 2019). However, age was also associated with lower biomechanical factors, possibly reflecting a differential distribution of physical work between older and younger workers (Bodin et al. 2020). No association was observed between female gender and shoulder disorders. This was not expected (Larsson et al. 2007), but female workers were less exposed to biomechanical factors, in accordance with the literature (Parent-Thirion et al. 2017). The association between overweight/obesity and shoulder disorders was of borderline significance; the literature being inconsistent regarding the association between this factor and shoulder pain/disorders (de Kok et al. 2019).

Sensitivity analyses were stratified by gender to take into account differences in the prevalence of exposure to workplace risk factors between men and women (Parent-Thirion et al. 2017). Overall, results were comparable between men and women, with a direct association between biomechanical factors and shoulder disorders and a lack of direct association between psychosocial factors and shoulder disorders. However, some differences were observed for the associations between organisational and psychosocial factors. These differences can be explained by different exposure to organisational and psychosocial factors between men and women (Parent-Thirion et al. 2017) due to the gender division of work, and also by the lack of statistical power due to the low number of cases.

The main strength of this study was its prospective design. In addition, shoulder disorder was assessed clinically by trained occupational physicians according to the recommendations of the European consensus criteria document for the evaluation of UEMSDs (Sluiter et al. 2001), enabling more accurate diagnosis of shoulder disorder than shoulder pain questionnaires, despite the fact that the protocol did not follow the recent recommendations (Papadonikolakis et al. 2011; Hermans et al. 2013). The diagnosis was uniquely based on clinical signs, because it was impossible to perform imaging in the occupational health setting. The relatively low incidence of shoulder disorders could be explained by a healthy worker effect in the regional working population, the most severe cases of shoulder disorders are not able to work.

SEM was used to study the direct and indirect relationships between occupational factors and shoulder disorders; this was not possible with classical logistic regression. SEM helps to explore relationships between risk factors and identify their respective direct and indirect roles. However, the hypotheses tested are based on a causal theoretical model. The current study had some limitations. The low number of cases of shoulder disorder could affect the statistical power of the study. In addition, the percentage of subjects lost to follow-up was high (56.6%). The follow-up rate for the second physical examination did not differ with gender or initial occupational category. The lowest participation rate was observed for young workers, those with an initial temporary job status, or those with a low length of service in their initial job. This was expected, but it was increased by the economic crisis in 2008, which strongly affected temporary employment, young workers and male workers. In addition, this could be explained by the difficulties of a longitudinal design in occupational medicine in France, related to the high mobility of both the occupational physicians and the workers (Sérazin et al. 2014).

A further limitation was the use of self-reported measures for the exposure variables, that may have increased the risk of common method variance (Podsakoff et al. 2003). However, standardised questionnaires were used. Biomechanical factors were based on the recommendations of the European consensus criteria for the evaluation of UEMSDs (Sluiter et al. 2001). Psychosocial factors were assessed using the French version of the JCQ (Niedhammer et al. 2006). Finally, the questions regarding a work pace dependent on an automatic rate and a work pace dependent on customers’ demands were taken from large-scale French studies conducted by the French Ministry of Labour (DARES) (Coutrot et al. 2018).

As with previous studies on shoulder pain and CTS conducted as part of the Cosali study (Bodin et al. 2018, 2020; Roquelaure et al. 2020), this study identified the complex direct and indirect relationships between occupational factors and shoulder disorders. Our data confirmed the conceptual causation model: organisational and psychosocial factors were associated with biomechanical factors, while biomechanical factors were linked to the incidence of shoulder disorders. It is necessary to improve the understanding of the pathways between determinants of UEMSDs. Organisational factors are an important target for interventions aiming to prevent shoulder disorders in the working population. Future research should include researchers from various disciplines (ergonomics, epidemiology, etc.) to determine which measures are effective in preventing shoulder disorders.

## Supplementary Information

Below is the link to the electronic supplementary material.

Supplementary file1 (DOCX 43 kb)

## Data Availability

The data sets analysed during the current study are available from the corresponding author on reasonable request.
