# Supplementary material for: Risk factors for shoulder disorders among French workers: prospective cohort study
Source: Int Arch Occup Environ Health. 2022 Mar 16;95(7):1511–9. doi: 10.1007/s00420-022-01853-9 (PMC9424162; doi:10.1007/s00420-022-01853-9)
Supplement: Supplementary file 1 — Supplementary file1 (DOCX 43 kb) [file 420_2022_1853_MOESM1_ESM.docx]

Appendix 1. Comparison of baseline characteristics of workers with follow-up and workers without follow-up (n = 3,710)

|  | **With follow-up (n=1,611)** | | **Without follow-up (n=2,099)** | | **p-value** |
| --- | --- | --- | --- | --- | --- |
|  | **n** | **%** | **n** | **%** |  |
| Gender | | | | | 0.243 |
| Male | 921 | 57.2 | 1240 | 59.1 |  |
| Female | 690 | 42.8 | 859 | 40.9 |  |
| Age (years) |  |  |  |  | **<0.001** |
| <35 | 533 | 33.1 | 892 | 42.5 |  |
| 35-44 | 568 | 35.3 | 492 | 23.5 |  |
| ≥45 | 510 | 31.7 | 714 | 34.0 |  |
| Overweight/obesity | 579 | 36.5 | 799 | 38.6 | 0.199 |
| Seniority in current job (years) |  |  |  |  | **<0.001** |
| <1 | 130 | 8.1 | 325 | 15.7 |  |
| 1-2 | 235 | 14.7 | 356 | 17.2 |  |
| 3-10 | 573 | 35.9 | 665 | 32.0 |  |
| >10 | 660 | 41.3 | 729 | 35.1 |  |
| Temporary employment | 93 | 5.8 | 333 | 15.9 | **<0.001** |
| Occupational category |  |  |  |  | 0.055 |
| Craftsmen, salesmen and managers | 6 | 0.4 | 10 | 0.5 |  |
| Professionals | 114 | 7.1 | 174 | 8.3 |  |
| Technicians, associate professionals | 378 | 23.5 | 451 | 21.5 |  |
| Low-grade white-collar workers | 455 | 28.3 | 531 | 25.3 |  |
| Blue-collar workers | 656 | 40.8 | 930 | 44.4 |  |
| Economic sector |  |  |  |  | **<0.001** |
| Agriculture | 13 | 0.8 | 58 | 2.8 |  |
| Industry | 599 | 37.2 | 623 | 29.7 |  |
| Construction | 80 | 5.0 | 134 | 6.4 |  |
| Trade and services | 918 | 57.0 | 1,282 | 61.1 |  |
| In bold, p-value < 0.05. | | | | | |

Appendix 2. Flow-chart of the study participants (n = 1,320)

Enrolled participants: N = 3,710

• Personal factors

• Nordic questionnaire

• Assessment of work-related factors

• Physical examination

**Follow-up**

**2007-2010**

**Baseline**

**2002-2005**

*Reason for non-participants: N = 1,228*

- 23 deaths
- 162 retired
- 17 on parental leave or long-term sick leave
- 105 usually watched by a non-participant occupational physician
- 921 lost to follow-up by their occupational physician due to loss of job

Participants surveyed by an occupational physician of the surveillance program: N = 2,482

Subjects for analysis: N = 1,320

*Reason for exclusion: N = 291*

- 6 craftsmen, salesmen and managers + 2 missing value at baseline
- 13 in the agriculture sector + 1 missing value at baseline
- 148 with shoulder disorder
- 121 with missing data on covariates

Participants seen during a regularly scheduled mandatory health

Examination: N = 1,611

*Reason for exclusion: N = 871*

- 848 not examined for the protocol
- 23 refusals to participate in the follow-up

Appendix 3. Results from structural equation modeling of relations between organisational, psychosocial, biomechanical, and personal factors shoulder disorders in French workers, Cosali (COhorte des SAlariés Ligériens) survey (N = 1,320)

|  | **Together (N=1,320, n=86)** | | | **Men (N=775, n=46)** | | | **Women (N=545, n=40)** | | |
| --- | --- | --- | --- | --- | --- | --- | --- | --- | --- |
|  | **Standardized beta** | **Standard error** | **p-value** | **Standardized beta** | **Standard error** | **p-value** | **Standardized beta** | **Standard error** | **p-value** |
| **Predictors of biomechanical exposure** |  |  |  |  |  |  |  |  |  |
| Work pace dependent on automatic rate | 0.21 | 0.03 | **<0.001** | 0.20 | 0.05 | **<0.001** | 0.26 | 0.05 | **<0.001** |
| Work pace dependent on customers’ demand | -0.06 | 0.04 | 0.114 | -0.07 | 0.05 | 0.157 | -0.03 | 0.06 | 0.561 |
| Decision authority | -0.07 | 0.04 | 0.079 | -0.09 | 0.05 | 0.096 | -0.06 | 0.06 | 0.371 |
| Skill discretion | -0.15 | 0.04 | **<0.001** | -0.09 | 0.06 | 0.104 | -0.21 | 0.06 | **0.001** |
| Psychological demand | 0.09 | 0.03 | **0.005** | 0.11 | 0.04 | **0.010** | 0.06 | 0.05 | 0.259 |
| Age≥45 | -0.08 | 0.03 | **0.027** | -0.08 | 0.05 | 0.097 | -0.08 | 0.05 | 0.124 |
| Female gender | -0.12 | 0.03 | **<0.001** |  |  |  |  |  |  |
|  |  |  |  |  |  |  |  |  |  |
| **Predictors of decision authority** |  |  |  |  |  |  |  |  |  |
| Work pace dependent on automatic rate | -0.17 | 0.02 | **<0.001** | -0.14 | 0.03 | **<0.001** | -0.23 | 0.03 | **<0.001** |
| Work pace dependent on customers’ demand | 0.18 | 0.03 | **<0.001** | 0.22 | 0.03 | **<0.001** | 0.14 | 0.04 | **0.001** |
| Female gender | -0.14 | 0.03 | **<0.001** |  |  |  |  |  |  |
|  |  |  |  |  |  |  |  |  |  |
| **Predictors of skill discretion** |  |  |  |  |  |  |  |  |  |
| Work pace dependent on automatic rate | -0.21 | 0.02 | **<0.001** | -0.20 | 0.03 | **<0.001** | -0.24 | 0.04 | **<0.001** |
| Work pace dependent on customers’ demand | 0.20 | 0.03 | **<0.001** | 0.20 | 0.03 | **<0.001** | 0.20 | 0.04 | **<0.001** |
| Female gender | -0.17 | 0.03 | **<0.001** |  |  |  |  |  |  |
|  |  |  |  |  |  |  |  |  |  |
| **Predictors of psychological demand** |  |  |  |  |  |  |  |  |  |
| Work pace dependent on automatic rate | 0.02 | 0.03 | 0.504 | 0.00 | 0.04 | 0.938 | 0.07 | 0.05 | 0.153 |
| Work pace dependent on customers’ demand | 0.19 | 0.03 | **<0.001** | 0.17 | 0.04 | **<0.001** | 0.23 | 0.04 | **<0.001** |
| Female gender | 0.04 | 0.03 | 0.178 |  |  |  |  |  |  |
|  |  |  |  |  |  |  |  |  |  |
| **Predictors of supervisor social support** |  |  |  |  |  |  |  |  |  |
| Work pace dependent on automatic rate | -0.05 | 0.03 | 0.072 | 0.01 | 0.03 | 0.717 | -0.16 | 0.04 | **<0.001** |
| Work pace dependent on customers’ demand | 0.01 | 0.03 | 0.814 | 0.02 | 0.04 | 0.495 | -0.03 | 0.04 | 0.533 |
| Female gender | 0.03 | 0.03 | 0.316 |  |  |  |  |  |  |
|  |  |  |  |  |  |  |  |  |  |
| **Predictors of coworkers social support** |  |  |  |  |  |  |  |  |  |
| Work pace dependent on automatic rate | -0.07 | 0.03 | **0.007** | -0.05 | 0.04 | 0.129 | -0.11 | 0.04 | **0.009** |
| Work pace dependent on customers’ demand | 0.06 | 0.03 | **0.027** | 0.11 | 0.04 | **0.002** | -0.01 | 0.04 | 0.884 |
| Female gender | 0.01 | 0.03 | 0.632 |  |  |  |  |  |  |
|  |  |  |  |  |  |  |  |  |  |
| **Predictors of shoulder disorder at follow-up** |  |  |  |  |  |  |  |  |  |
| Biomechanical exposure | 0.20 | 0.07 | **0.004** | 0.17 | 0.09 | 0.051 | 0.20 | 0.09 | **0.023** |
| Decision authority | -0.05 | 0.06 | 0.411 | 0.05 | 0.08 | 0.555 | -0.13 | 0.08 | 0.131 |
| Skill discretion | 0.10 | 0.07 | 0.122 | 0.07 | 0.10 | 0.458 | 0.13 | 0.09 | 0.141 |
| Psychological demand | 0.03 | 0.04 | 0.463 | 0.13 | 0.07 | 0.052 | -0.07 | 0.06 | 0.238 |
| Supervisor social support | -0.04 | 0.06 | 0.487 | -0.05 | 0.09 | 0.570 | -0.04 | 0.08 | 0.629 |
| Coworkers social support | -0.03 | 0.05 | 0.612 | -0.10 | 0.08 | 0.197 | 0.02 | 0.08 | 0.806 |
| Age≥45 | 0.24 | 0.05 | **<0.001** | 0.22 | 0.07 | **0.001** | 0.27 | 0.07 | **<0.001** |
| Overweight / Obesity | 0.09 | 0.05 | 0.071 | 0.06 | 0.08 | 0.439 | 0.13 | 0.07 | 0.071 |
| Female gender | 0.09 | 0.06 | 0.117 |  |  |  |  |  |  |
|  |  |  |  |  |  |  |  |  |  |
| **Correlations** |  |  |  |  |  |  |  |  |  |
| Biomechanical exposure /Supervisor social support | -0.05 | 0.03 | 0.108 | -0.05 | 0.04 | 0.223 | -0.04 | 0.05 | 0.374 |
| Biomechanical exposure /Coworkers social support | 0.00 | 0.03 | 0.907 | -0.04 | 0.04 | 0.414 | 0.03 | 0.06 | 0.549 |
|  |  |  |  |  |  |  |  |  |  |
| Decision authority/Skill discretion | 0.50 | 0.02 | **<0.001** | 0.52 | 0.02 | **<0.001** | 0.47 | 0.03 | **<0.001** |
| Decision authority/Psychological demand | 0.00 | 0.02 | 0.955 | 0.01 | 0.03 | 0.809 | 0.00 | 0.04 | 0.994 |
| Decision authority/Supervisor social support | 0.22 | 0.02 | **<0.001** | 0.24 | 0.03 | **<0.001** | 0.18 | 0.04 | **<0.001** |
| Decision authority/Coworkers social support | 0.12 | 0.02 | **<0.001** | 0.19 | 0.03 | **<0.001** | 0.03 | 0.04 | 0.385 |
|  |  |  |  |  |  |  |  |  |  |
| Skill discretion/Psychological demand | 0.20 | 0.02 | **<0.001** | 0.21 | 0.03 | **<0.001** | 0.19 | 0.04 | **<0.001** |
| Skill discretion/Supervisor social support | 0.27 | 0.02 | **<0.001** | 0.27 | 0.03 | **<0.001** | 0.28 | 0.03 | **<0.001** |
| Skill discretion/Coworkers social support | 0.15 | 0.02 | **<0.001** | 0.23 | 0.03 | **<0.001** | 0.06 | 0.04 | 0.127 |
|  |  |  |  |  |  |  |  |  |  |
| Psychological demand /Supervisor social support | -0.12 | 0.02 | **<0.001** | -0.11 | 0.03 | **<0.001** | -0.15 | 0.04 | **<0.001** |
| Psychological demand/Coworkers social support | -0.04 | 0.02 | 0.121 | 0.02 | 0.03 | 0.519 | -0.11 | 0.04 | **0.007** |
|  |  |  |  |  |  |  |  |  |  |
| Supervisor social support/Coworkers social support | 0.33 | 0.02 | **<0.001** | 0.28 | 0.02 | **<0.001** | 0.40 | 0.02 | **<0.001** |
| In bold, p-value < 0.05. | | | | | | | | | |
